# Structural insights of human N-acetyltransferase 10 and identification of its potential novel inhibitors

**DOI:** 10.1038/s41598-021-84908-0

**Published:** 2021-03-15

**Authors:** Mahmood Hassan Dalhat, Hisham N. Altayb, Mohammad Imran Khan, Hani Choudhry

**Affiliations:** 1 Biochemistry Department, Faculty of Science, King Abdulaziz University, Jeddah, Kingdom of Saudi Arabia; 2 Centre for Artificial Intelligence in Precision Medicine, King Abdulaziz University, Jeddah, Kingdom of Saudi Arabia; 3 Cancer and Mutagenesis Research Unit, King Fahd Medical Research Center, King Abdulaziz University, Jeddah, Kingdom of Saudi Arabia

**Keywords:** Virtual drug screening, Epigenomics

## Abstract

N-acetyltransferase 10 (NAT10), is an acetyltransferase that regulates RNA stability and translation processes. Association of NAT10 with several diseases including cancer, makes it a promising therapeutic target. Remodelin is the only known NAT10 inhibitor, but the structural information related to its binding with NAT10 is still obscure. Here, we predicted the human NAT10 structure using homology modeling that was not available previously and used human NAT10 to identify the novel binding site(s) of Remodelin. The alignment of the modeled human NAT10 showed 24% identity and 37% positivity with crystal structure of tRNA (Met) cytidine acetyltransferase. Molecular docking showed binding of Remodelin with NAT10 in acetyl-CoA binding pocket. Additionally, we screened a library of FDA-approved drugs for the identification of novel inhibitors of NAT10 activity. Binding score showed that four drugs namely, Fosaprepitant (− 11.709), Leucal (− 10.46), Fludarabine (− 10.347) and Dantrolene (− 9.875) bind to NAT10 and have better binding capability when compared with Acetyl-CoA (− 5.691) and Remodelin (− 5.3). Acetyl-CoA, Remodelin, and others exhibit hits for hydrophobic, hydrophilic and hydrogen interactions. Interestingly, Remodelin and others interact with the amino acid residues ILE629, GLY639, GLY641, LEU719, and PHE722 in the Acetyl-CoA binding pocket of NAT10 similar to Acetyl-CoA. Our findings revealed that Fosaprepitant, Leucal, Fludarabine, and Dantrolene are promising molecules that can be tested and developed as potential inhibitors of NAT10 acetyltransferase activity.

## Introduction

N-acetyltransferase 10 (NAT10) is a nucleolar protein and a member of the GNAT family of KATs. NAT10 is known to add an acetyl group(s) to RNAs^1^, and proteins such as p53^2^, Che-1, α-tubulin, poly(ADP-ribose) polymerase 1 (PARP1), and MORC2^3^. Previous studies have identified NAT10 as a regulator of cell activities, such as RNA acetylation, ribosome biogenesis, transcription, and translation. Focusing on RNA acetylation, NAT10 is identified as the writer of N4 acetylcytidine (ac^4^C) known to stabilize RNA translational efficiency by adding acetyl groups at the 5ʹUTR and coding regions as shown by transcriptome-wide ac^4^C quantitative mapping in rRNA, tRNA and mRNA^1,4,5^. The dynamic landscape of ac^4^C distribution across RNA molecules and its function in translational efficiency could be why several cancer features and systemic lupus erythematosus (SLE) are associated with NAT10^5,6^.

Deregulation of NAT10 is seen in Hutchinson-Gilford progeria syndrome, human immunodeficiency virus (HIV), and various cancer types^7–9^. Overexpression of NAT10 is shown to promote cell proliferation in cells expressing mutant p53 by counteracting the MDM2 activity^10^. It is also shown to regulate p53 by acetylation^2^. NAT10 promotes cell cycle progression; studies involving NAT10 knockdown and inhibition showed silencing NAT10 result in cell cycle arrest by prolonging the S phase. The proposed mechanism via which NAT10 promotes cell cycle progression is via the cyclinD1/CDK2/p21 axis^11^. NAT10 is shown to promote DNA damage response; this is confirmed by increase in NAT10 protein and gene expression levels upon treatment with genotoxic agents such H_2_O_2_ and cisplatin^12^. In support of the role of NAT10 in mediating DNA damage response, two studies involving MORC family CW-type zinc finger 2 (MORC2) showed NAT10 regulates DNA repair and cell survival by acetylation of MORC2 thereby preventing PARP1 degradation by E3 ubiquitin ligase CHFR via NAT10/MORC2/PARP1 axis of the PARP1 dependent pathway^13^. Similarly, NAT10 regulates cell cycle checkpoint and prevent DNA damage by acetylation of MORC2 by mediating cell cycle arrest and cell survival through NAT10/MORC2/H3T11P/cyclinB1/CDK1 axis^3^.

NAT10 is reported to be involved in cell migration through the CREB/MYCT1/NAT10 axis. This is evidently shown by synergistic expression of CREB and NAT10 in larynphageal cancer. The CREB is known to be involved in cell migration and metastasis through the FAK/CREB/TNNC1 axis. NAT10 is reported to regulate hypoxia-induced epithelial to mesenchymal transition (EMT) by regulating the expression of EMT biomarkers such as E-cadherin, vimentin, snail, and slug^14^. Interestingly, another study showed inhibition of NAT10 could attenuate doxorubicin resistance by reversing the effect of EMT biomarkers twist and vimentin^15^.

Several evidences have pointed to the role of NAT10 in cell proliferation, cell migration, DNA damage response, and EMT suggests that NAT10 could be a suitable therapeutic target for cancer treatment^7,14,16–18^. Remodelin, a small-molecule compound, has already been explored as potent NAT10 inhibitor^7^. It is shown to have a reverse effect on cell proliferation, cell invasion, and migration under hypoxic conditions by blocking the epithelial-mesenchymal transition (EMT)^14,19^. Remodelin is also reported to attenuate doxorubicin resistance in hepatocellular carcinoma and breast cancer by reducing EMT expression and hypoxia biomarkers^15^. The expression levels of EMT biomarkers E-caherin and vimentin were shown to be altered, favoring epithelial restoration upon the treatment with Remodelin. Furthermore, in vivo and in vitro studies in both hepatocellular carcinoma and breast cancer showed an increase in sensitivity of cancer cells to doxorubicin upon the addition of Remodelin. Hypoxic conditions in cancer reported being suppressed in cancer cells treated with Remodelin. This was confirmed after inducing hypoxia using CoCl_2_ followed by treatment with Remodelin. The expression level of NAT10 as well as HIF1α and HIF2α, were decreased^17^. The role of Remodelin as a regulator of EMT and hypoxia were all confirmed using NAT10 siRNA. In melanoma, Remodelin was reported to repress microphthalmia-associated transcription factor (MITF) required for melanogenesis and melanoma growth^11^.

Although many studies have recently provided evidence that Remodelin treatment reduced NAT10 expression and mRNA acetylation, it is still unclear how and where Remodelin binds with NAT10 protein. In the current study, we have provided the novel structural insights of Remodelin-NAT10 interaction by using the human NAT10 structure, which was not available earlier. We have also used the predicted human NAT10 structure to screen novel FDA-approved drugs for repurposing as inhibitors of NAT10 activity.

## Results

### Homology modeling and model quality of human NAT10

**Figure 1:**
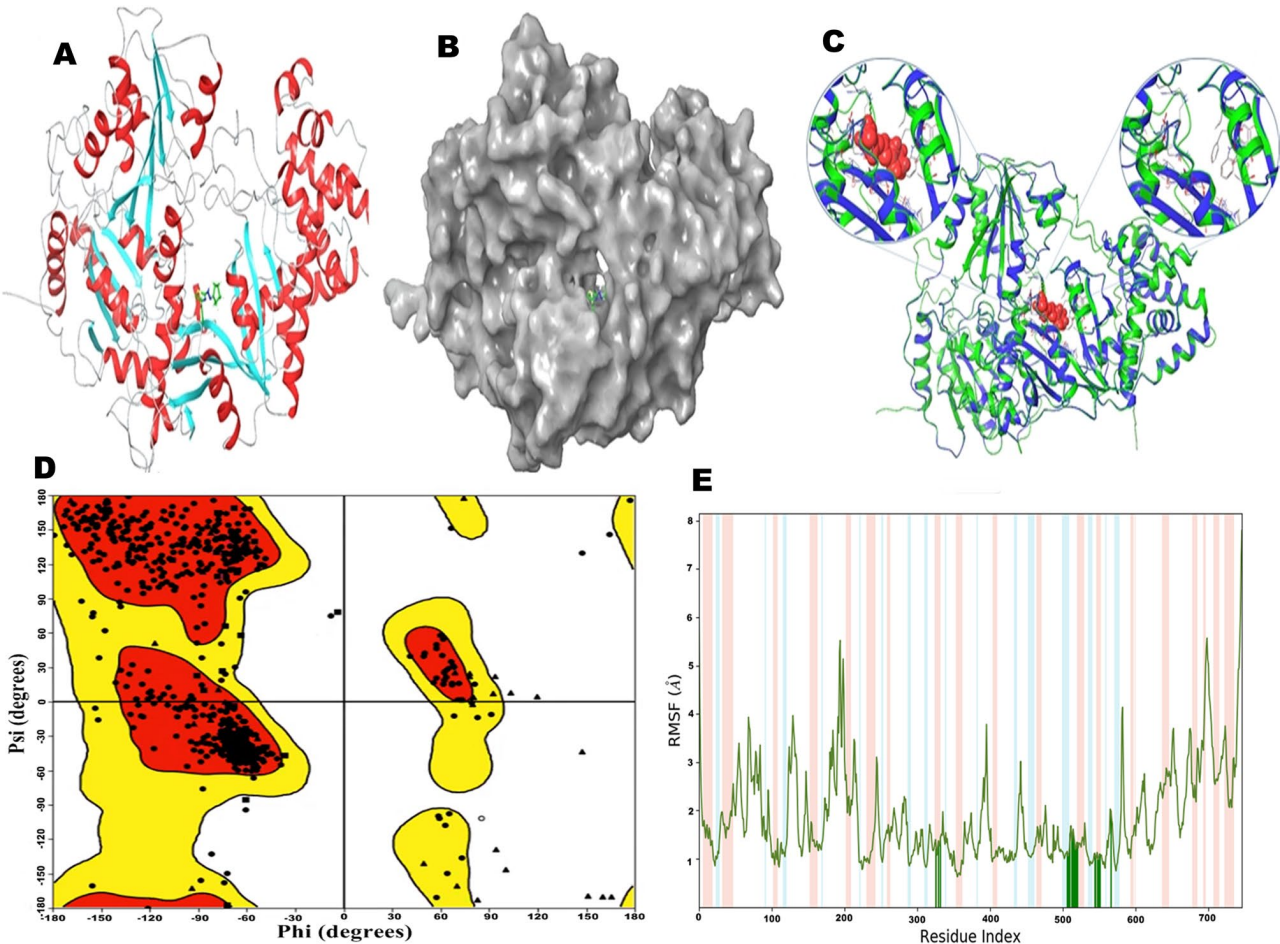
3D structure of human NAT10 generated model with Remodelin. (**A**) Model chains of human NAT10 in different colors with Remodelin in its active site. (**B**) Solid model of human NAT10 showing the active site groove containing Remodelin. (**C**) Superposition of the human NAT10 model (green) and the structure of template tRNA (Met) Cytidine Acetyltransferase (PDB ID: 2ZPA) (white), with enlarged active site pocket and residues interacting with Remodelin. (**D**) Ramachandran plot of human NAT10 generated model. Red and yellow are allowed regions. Triangles indicate glycine, squares are proline, circles indicate other amino acid residues on the protein. (**E**) The Root Mean Square Fluctuation (RMSF) plot of human NAT10 protein, peaks indicate the fluctuation of human NAT10 during simulation, and the green-colored vertical bars indicate acetyl-CoA binding site residues interacting with Remodelin.

The human NAT10 is not available in the protein data bank (PDB) at the time of the study, so we used primary protein sequences with 1025 amino acids retrieved from UniProt (NP_078938.3, UniProt ID: QH0H0), to build the human NAT10 protein model. The Prime tool of the Maestro interface (Schrodinger) was used for model generation (Fig. 1A). The generation of 3D structure begins with searching for homologous proteins using Primer interface; the alignment showed 24% identity and 37% positivity with crystal structure of tRNA (Met) Cytidine Acetyltransferase (PDB ID: 2ZPA) (Fig. 1B), that have a good resolution (2.35 Å). The residues and chains around the active site showed high similarity, as shown in Fig. 1C. Ramachandran plot showed most of the residues in the generated model in their allowed regions, few of proline and glycine were found outlier (Fig. 1D). Root mean square fluctuation (RMSF) showed that residues in the active site are of high stability during simulation time, as shown in Fig. 1E. Overall, both Ramachandran plot and RMSF showed that the modeled human NAT10 is stable and could be used for molecular docking.

### Remodelin like acetyl-CoA binds and interacts with the acetyl-CoA binding pocket of human NAT10

**Figure 2:**
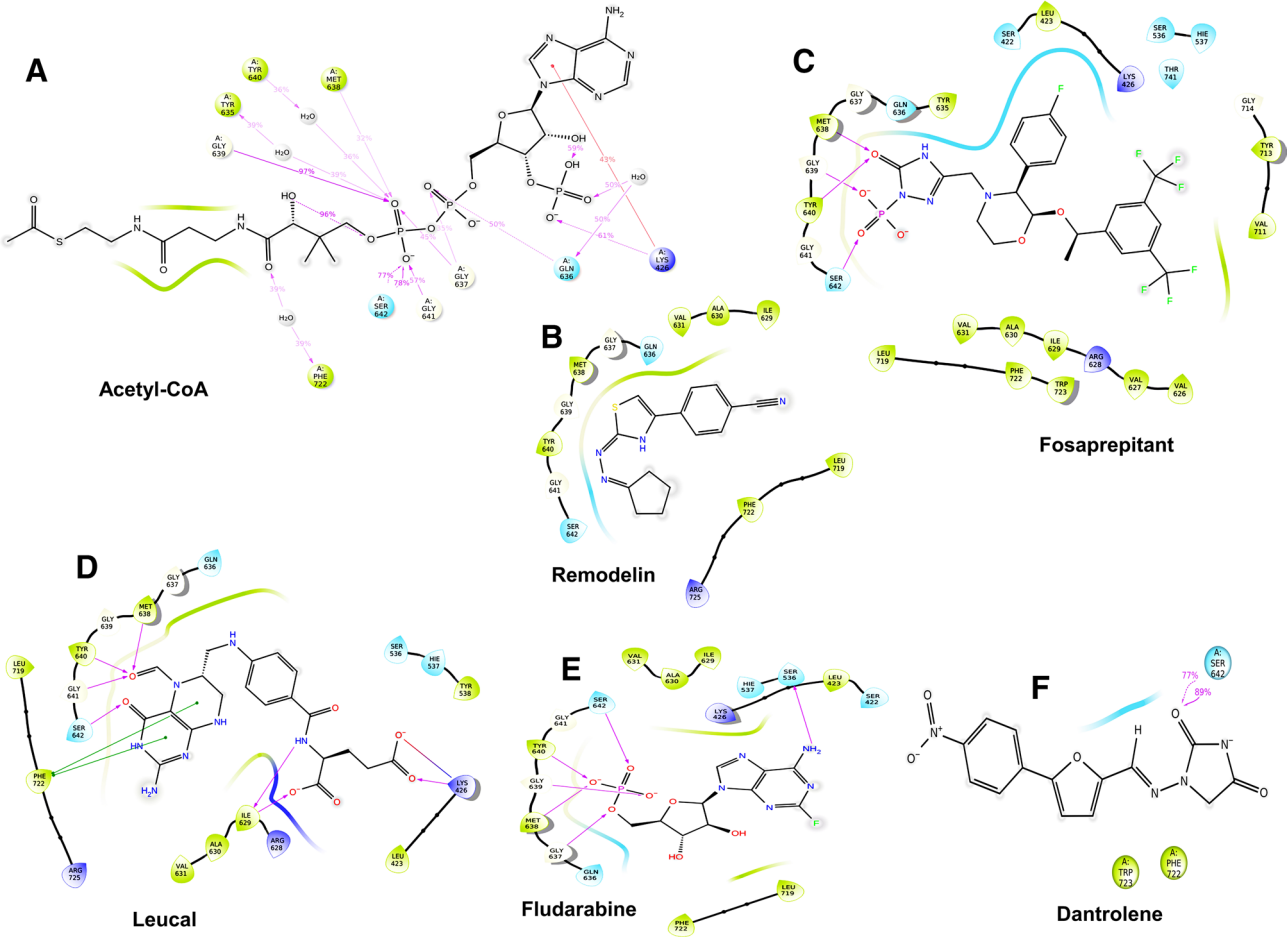
Interaction diagram of NAT10 residues from acetyl-CoA binding pocket with ligands. (**A**) Acetyl-CoA (**B**) Remodelin (**C**) Fosaprepitant (**D**) Leucal (**E**) Fludarabine (**F**) Dantrolene.

**Figure 3:**
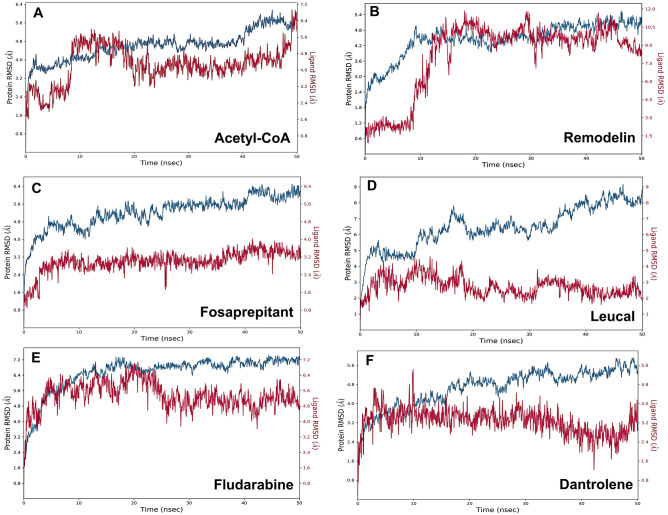
The Root Mean Square Deviation (RMSD) plots of ligands interacting with NAT10. RMSDs for (**A**) Acetyl-CoA (**B**) Remodelin (**C**) Fosaprepitant (**D**) Leucal (**E**) Fludarabine (**F**) Dantrolene. Blue colour represents NAT10 backbone fluctuations; red represents ligand fluctuations.

Ligand docking findings and MD simulation of Acetyl-CoA (substrate of NAT10) provided sufficient information on binding affinity, stability and orientation of Acetyl-CoA-NAT10 interactions which could be the basis for Remodelin inhibition of human NAT10 as illustrated in Figs. 2A and 3A. Acetyl-CoA was observed to be stable with NAT10 backbone throughout MD simulation of 50ns (Fig. 3A).

**Figure 4:**
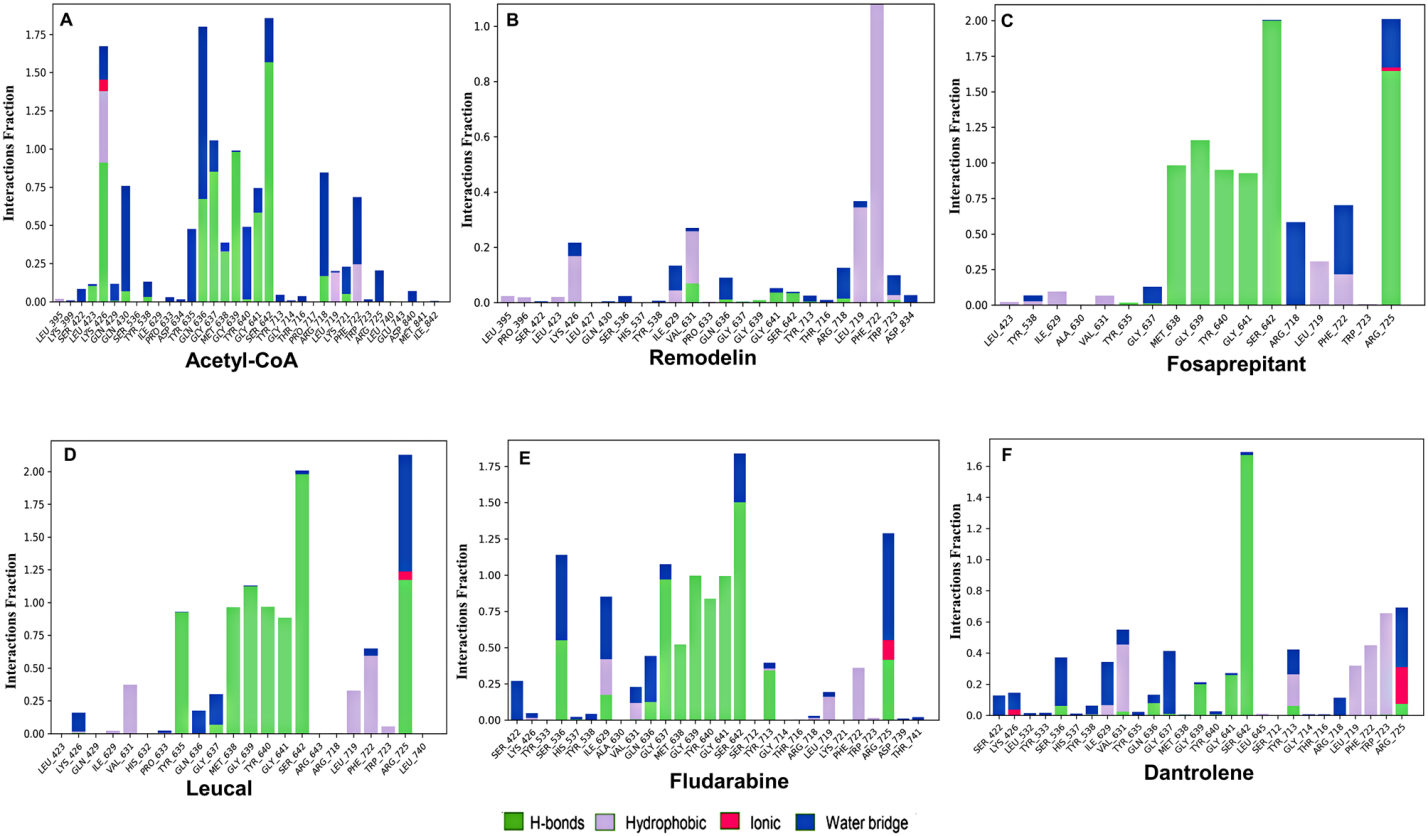
The NAT10-Ligands contacts showing the bonding interactions fraction. (**A**) Acetyl-CoA (**B**) Remodelin (**C**) Fosaprepitant (**D**) Leucal (**E**) Fludarabine (**F**) Dantrolene.

Remodelin docked NAT10 revealed stable binding and interaction of Remodelin with the acetyl-CoA binding pocket (Figs. 2B, 3B). The NAT10 bound-Remodelin contains glide score of − 5.3, docking score of − 4.96 and glide energy of − 38.93 kcal/mol (Table 1) and exhibits good binding state during MD simulation. The plot of Remodelin during RMSD analysis was observed to be stable by aligning with the NAT10 backbone (Fig. 3B). Remodelin fluctuated at the first 15 ns simulation time and then was stable with NAT10 backbone until 45 ns (Fig. 3B). During the whole MD simulation analysis, the NAT10-Remodelin complex was stable, indicating the binding stability of Remodelin to NAT10. Hydrophobic and hydrogen bond interactions were noticed between Remodelin and amino residues of NAT10 including, lysine (LYS426), valine (VAL631), leucine (LEU719) and phenylalanine (PHE722) (Fig. 4B). The PHE722 exhibited π–π stacking interaction (33%) during the MD simulation. Next, we compared the results obtained from both acetyl-CoA/NAT10 and Remodelin/NAT10 interactions. Surprisingly, the docking score and glide score of Acetyl-CoA/NAT10 interaction are similar to Remodelin/NAT10 interaction, which are − 4.36 and − 4.96 respectively for docking score and − 5.96 and − 5.3 respectively for glide score (Table 1). However, the glide energy of acetyl-CoA/NAT10 interaction is higher (− 77.94 kcal/mol) compared to Remodelin/NAT10 (− 38.93 kcal/mol). Molecular interactions revealed similarities in acetyl-CoA- and Remodelin-docked NAT10 in amino acid residues such as LYS426, GLN639, GLY639, GLY641, ARG718, LEU719, and PHE722 (Fig. 4A,B).

Similarities in the molecular interaction pattern between Acetyl-CoA- and Remodelin-docked NAT10 agree with several studies that showed Remodelin could be a potent inhibitor of NAT10^7,8,11,17^. Based on the NAT10-bound Remodelin complex, we then screened FDA-approved drugs for possible inhibitors of NAT10.

**Table 1:** Acetyl-CoA, Remodelin and top 20 shortlist drugs by SP-docking for targeting NAT10.

S. no.	Molecule	XP GScore	Docking score	Glide Gscore	Glide energy (kcal/mol)	Current therapeutic indications
1	Acetyl-CoA	− 5.691	− 4.355	− 5.691	− 77.944	Substrate for NAT10 mediated acetylation
2	Remodelin	− 5.3	− 4.96	− 5.3	− 38.93	Small molecule for NAT10 inhibition
3	Fosaprepitant	− 11.709	− 11.704	− 11.709	− 65.754	Anti-emetic drug
4	Leucal	− 10.46	− 10.46	− 10.46	− 68.304	Anti-neoplatic adjuct and antidote to folic acid agonist
5	Fludarabine	− 10.347	− 10.255	− 10.347	− 51.163	For the treatment of leukemia and lymphoma
6	Dantrolene	− 9.875	− 8.791	− 8.98	− 38.959	Postsynaptic muscle relaxant
7	Diphosphoric acid	− 9.543	− 8.594	− 9.875	− 42.637	E. coli metabolite
8	Risedronate	− 8.98	− 8.842	− 9.543	− 45.774	For treatment of osteoporosis
9	Cidovir	− 8.918	− 8.365	− 8.918	− 45.691	For the treatment of cytomegalovirus retinitis in HIV patients
10	Pemetrexed	− 8.361	− 7.912	− 8.498	− 47.026	For the treatment of lung cancer and mesothelioma
11	Ceftriaxone	− 8.345	− 8.361	− 8.361	− 52.927	Antibiotic
12	Balsalazide	− 8.152	− 8.345	− 8.345	− 68.626	Anti-inflammatory for treament of bowel disease
13	Zoledronate	− 8.335	− 7.791	− 8.335	− 41.21	For treatment of bone diseases i.e. Osteoporosis
14	Fospropofol	− 8.167	− 8.109	− 8.167	− 38.095	Sedative
15	Lifitegrast	− 8.015	− 8.151	− 8.152	− 53.717	Anti-inflammatory for treament of karatoconjunctivitis
16	Teriflunomide	− 8.011	− 6.751	− 8.032	− 35.784	For treatment of multiple sclerosis
17	Iohexol	− 7.935	− 8.015	− 8.015	− 67.566	Use as radiographic contrast medium
18	Tenofovir (tdf)	− 8.498	− 8.006	− 8.011	− 41.006	For treatment of HIV
19	Folotyn	− 7.944	− 7.903	− 7.944	− 51.167	For managing relapse and refractory peripheral T-cell lymphoma
20	Lipitor	− 7.83	− 7.935	− 7.935	− 62.216	Statin medication for cardiovascular disease
21	Zoledronate	− 8.335	− 7.827	− 7.83	− 63.855	For treatment of osteoporosis and high Ca^2+^ due to cancer
22	Ioxilan	− 7.64	− 7.64	− 7.64	− 60.687	Use as radiographic contrast medium

### Virtual screening of FDA approved drugs for identification of novel human NAT10 inhibitors

A list of 2115 US-FDA-approved drugs were screened for their inhibitory effect on NAT10 protein, 200 molecules with the best docking score (ranged from − 5 to − 11.7), were selected for SP-docking, from which the best 50 molecules were screened using XP-docking. The top four (4) screened FDA-approved drugs were Fosaprepitant (Fig. 2C); an anti-emetic, has glide score of − 11.7, followed by Leucal (Fig. 2D), an antineoplastic and antidote to folic acid agonist with glide score of − 10.5, then Fludarabine (Fig. 2E), which is used for the treatment of leukemia and lymphoma, showed − 10.35 glide score and Dantrolene (Fig. 2F), approved as muscle relaxant has glide score of − 8.98 (Table 1). As observed the glide scores of the top 4 screened drugs are better than acetyl-CoA (− 5.69) and Remodelin (− 5.3), showing that the screened drug could be better inhibitors than Remodelin. To confirm our prediction of the top screened FDA-approved drugs, we further subjected the NAT10-bound complexes of Fosaprepitant, Leucal, Fludarabine and Dantrolene to MD simulation.

### Molecular dynamic simulations predict stable molecular interactions between Fosaprepitant, Leucal, Fludarabine and Dantrolene with human NAT10

**Figure 5:**
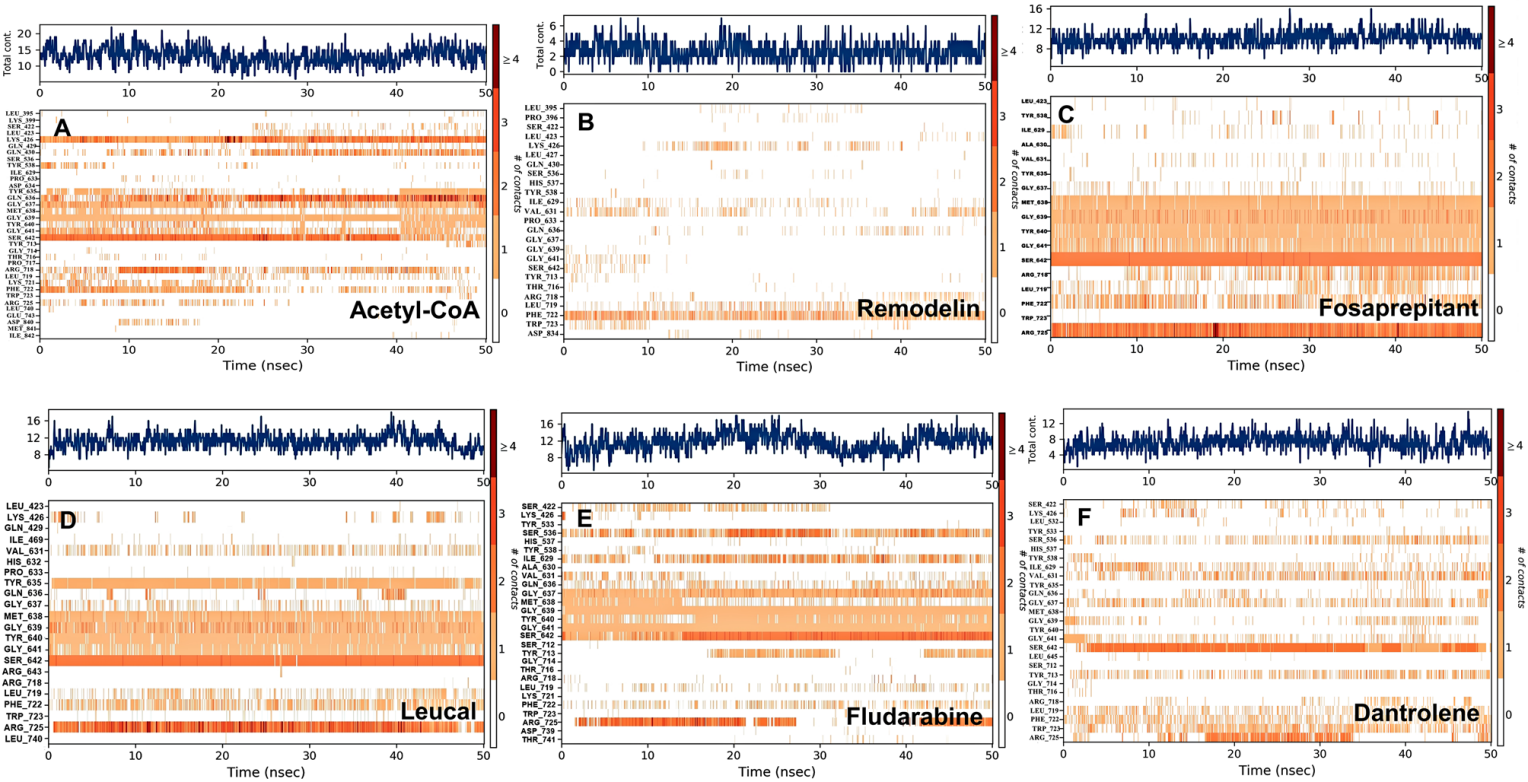
Interaction of each residue of the acetyl-CoA binding pocket with ligands in each trajectory frame. (**A**) Acetyl-CoA (**B**) Remodelin (**C**) Fosaprepitant (**D**) Leucal (**E**) Fludarabine (**F**) Dantrolene. Deeper coloration corresponds to more contact between ligands and NAT10.

MD simulation is used for the estimation of dynamics and stability of the protein-ligand complex. Four complexes with the best score and interaction from the docking were run at 50 ns simulation time, as shown in Fig. 3C–F. The simulation plots indicate the stability of protein after 10 ns simulation time with little fluctuation remain less 3Å indicates the stability of the generated model. The complex of NAT10 and Fosaprepitant showed high stability, the ligand RMSD stabilized shortly after 4 ns simulation time, with variation remained well below 1 Å, ranging between 3 and 4 Å during most time of simulation (Fig. 3C). The RMSD plot in Fig. 3D indicates the 50 ns trajectory of the complex of NAT10 and Leucal; a divergence was observed after 10 ns simulation time. However, the fluctuation lies under the acceptable range of 1–3 Å. In Fig. 3E the simulation showed that fludarabine was aligned with NAT10 backbone until 22 ns simulation time. However, the complex maintained its stability during the whole simulation time with a fluctuation of less than 3 Å. In Fig. 3F the simulation showed that dantrolene was aligned with NAT10 backbone until 15 ns simulation time, but it maintains stability throughout the 50ns MD simulation. The strongest molecular interaction between ligand and NAT10 amino residues was found at SER642 for Acetyl-CoA (Fig. 4A), PHE722 for Remodelin (Fig. 4B), ARG725 for Fosaprepitant (Fig. 4C), SER642 for Leucal (Fig. 4D), ARG725 for Fludarabine (Fig. 4E) and SER642 for Dantrolene (Fig. 4E). Molecular interactions such as VAL631, GLY639, GLY641, LEU719, and PHE722 are the most predominant form of interaction shared by Acetyl-CoA, Remodelin, Fosaprepitant, Leucal, Fludarabine and Dantrolene (Figs. 4 and 5). Interestingly, all the six ligands bind to GLY641 through hydrogen bond (Figs. 4 and 5). This showed that the position GLY641 is important for ligand binding on the acetyl-CoA binding site. Larrieu et al. have reported mutant NAT10 (G641E), although this mutation is shown not to affect Remodelin inhibitory function in laminopathic cells^7^. Based on the similarities in the molecular interaction sites between the compounds with acetyl-CoA binding pocket in the N-acetyltransferase domain of NAT10; we performed the molecular sequence alignment of the N-acetyltransferase domain in some organisms such as *Homo sapiens* (Q9H0A0), *Rattus norvegicus* (G3V752), *Mus musculus* (Q8K224), *Drosophila melanogaster* (Q9W3C1), *Escherichia coli* (P76562), *Schizosaccharomyces pombe* (P87115), *Arabidopsis thaliana* (Q9M2Q4), *Dictyostelium discoideum* (Q55EJ3), *Caenorhabditis elegans* (O01757), *Haemophilus influenzae* (P44140), *Vibrio cholerae serotype O1* (Q9KKJ5), *Salmonella typhimurium* (Q8ZN74), *Yersinia pestis* (D0JFM7), and *Yersinia enterocolitica serotype O:8* (A1JL12).

**Figure 6:**
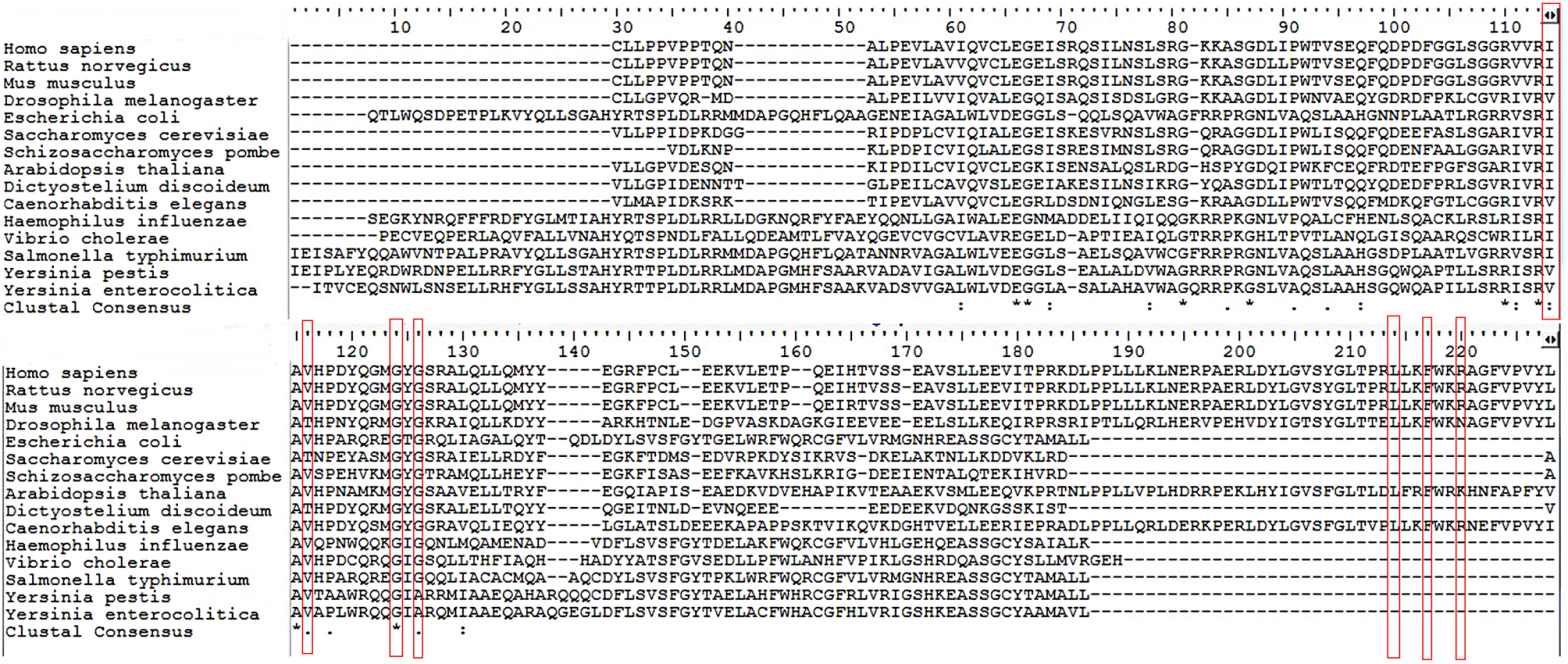
Multiple sequence alignment of the N-acetyltransferase domain showed multiple conserved regions in acetyl-CoA binding site. The most predominant amino acid residues that interact with all six (6) ligands are conserved as illustrated in red boxes.

Multiple sequence alignment of N-acetyltransferase domain of NAT10 from different organisms is shown in Fig. 6. We observed the most predominant amino acid residues which interact with all compounds including acetyl-CoA and Remodelin are in conserved regions. The conserved region found across aligned species are ILE629, VAL631, GLY639, and GLY641. The amino acid residue with 100% conservation across the studied species shown to bind with all studied drugs including acetyl-CoA and Remodelin is GLY639 which could play a significant role in future NAT10 activity studies. Overall, our findings suggest the important role of amino acid residues such as ILE629, VAL631, GLY639, and GLY641 in the activity of NAT10 and could be explored as target sites to develop novel inhibitors of NAT10.

## Discussion

Several studies have reported Remodelin as a potential NAT10 inhibitor, but the molecular architecture of interaction between Remodelin and human NAT10 is not established yet. Here we predicted the binding domain and interactions that are involved between Remodelin and human NAT10. Using the Remodelin docked hNAT10 complex, we further screened 2115 FDA-approved drugs to identify novel NAT10 inhibitors.

In NAT10, there are four (4) major domains identified to date. The N-acetyltransferase domain is responsible for acetylation of RNA, and proteins such as p53, MORC2, PARP, and α-tubulin using acetyl-CoA substrate^1–3,20^. Our molecular docking result showed stability of Remodelin molecule in the acetyl-CoA binding pocket through molecular docking and MD simulation results (Figs. 2B and 3B). Significant molecular interactions were detected between Remodelin and acetyl-CoA binding site on NAT10 at positions LYS426, VAL631, LEU719 and PHE722 (Fig. 4). Remodelin is presumed to prevent acetylation activity of NAT10 by occupying the acetyl-CoA binding pocket. In line with this prediction, Larrieu et al. reported decreased acetyltransferase activity upon treatment with Remodelin in an in vitro study conducted in Hutchinson-Gilford progeria syndrome (HGPS)^7^. A biophysical study by Shrimp et al. 2020 showed that Remodelin does not inhibit NAT10 dependent cytidine acetylation and could interact with multiple protein targets in the cell^21^. Contrary to this assertion, our predictive study is in line with Larrieu et al. showing Remodelin binds to the acetyl-CoA binding site, and could affect acetylation activity by preventing the proper binding of acetyl-CoA which is a substrate to RNA and protein acetylation^7^. Studies have shown Remodelin decreases NAT10 expression in HGPS^18,22^, HIV^8^ and cancer^9,15,17^. Physiologically, Remodelin is shown to ameliorate HGPS defects, reduce HIV copies, and attenuate EMT features of cancer. In addition, Remodelin was shown to weaken doxorubicin resistance and suppress hypoxia in cancer through NAT10/(VIM/TWIST/CDH1) and NAT10/(HIF1A/HIF2A) respectively^9,15,17^. Based on the potential of NAT10 as a therapeutic target in diseases such as cancer, HGPS and HIV, and the role of Remodelin as small molecule inhibitor of NAT10, we carried out virtual screening of FDA-approved drugs retrieved from Zinc database. In our study, we selected top 3 drugs base on docking score for MD simulation. We are the first to report these drugs as possible inhibitors of NAT10. These drugs include Fosaprepitant, Leucal (also referred to as Leucovorin), Fludarabine and Dantrolene (Table 1).

Fosaprepitant is a prodrug of aprepitant used as anti-emetic drug^23^. It aids in prevention of both delayed and acute nausea and vomiting associated with cancer chemotherapy^24^. Fosaprepitant is a neurokinin 1 receptor (NK-1R) antagonist that binds and deactivate NK-1R leading to downstream effect such as Ca^2+^ signaling through the g-protein coupled receptor (GPCR) cascade, which in turn leads to cellular sequestration resulting in vomiting^24–26^. Fosaprepitant exerts its effect on targeted cells by passing across the blood brain barrier (BBB) to its target destination^23^.

Leucal is an antineoplastic and antidote for folic acid antagonists^27^. It is a folate analog and is used to counteract the toxic effect of folate antagonists such as methotrexate^28,29^. Methotrexate is a potent anticancer drug that mechanistically targets dihydrofolate reductase^28^. Leucal and its analog are used as rescue therapy when there is a high dose of methotrexate in cancer treatment, such as osteosarcoma^30^. Leucal could be used in the treatment of megaloblast anemias due to folate deficiency.

Fludarabine belongs to a group of chemotherapeutic drugs referred to as anti-metabolites^31^. It is used to treat hematological malignancies such as acute lymphatic leukemia (ALL) and chronic lymphatic leukemia (CLL)^32,33^. Fludarabine phosphate usually dephosphorylates to 2-fluoro-ara-A and then phosphorylates intracellularly by deoxycytidine kinase to produce an active 2-fluoro-ara-ATP. The 2-fluoro-ara-ATP acts by inhibiting nucleotide biosynthesis enzymes such as the DNA polymerase alpha, ribonucleotide reductase, and DNA primase^34,35^. The role of fludarabine in nucleotide biosynthesis makes it an excellent anticancer drug^36–38^.

Dantrolene is a hydantoin derivative, but unlike other derivatives of hydantoin, it does not exhibit antiepileptic activity^39^. Dantrolene is an antagonist of Ryanodine receptor 1 (RyR1), which depresses excitation–contraction coupling in skeletal muscle^40,41^. RyR1 is known as a regulator of calcium ion (Ca^2+^) release from the sarcoplasmic reticulum during calcium signaling; this is an essential step in muscle contraction. As RyR1 antagonist, Dantrolene is used as drug for treating diseases related to muscular contraction such as spasticity and malignant hyperthermia^39,42–44^.

Based on our prediction, Fosaprepitant, Leucal, Fludarabine, and Dantrolene combined with other treatment regimens could improve cancer and HIV therapy. In cancer, inhibition of NAT10 is shown to increase DNA damage, decreased cell survival and cell cycle arrest; these properties could be explored to sensitize cancer cells to radiotherapy and chemotherapy by adding either of the screened drugs; Fosaprepitant, Leucal, Fludarabine and Dantrolene to the standard treatment regimen. The screened drugs could be effective in attenuating cancer drug resistance as already been reported in studies involving reversing doxorubicin resistance in hepatocellular carcinoma and breast cancer^9,15^. The screened drugs could also be explored in HIV treatment as it was reported that silencing or inhibiting NAT10 could reduce RNA viral stability, viral replication and viral copies^8^. Therefore, adding either of the screened drugs to the HIV treatment regimen could improve antiviral therapy.

Overall, our study here provides for the first time a structural insight of how and where Remodelin interacts with human NAT10. Further, we have also predicted a highly efficient human NAT10 structure that we used to screen FDA-approved library for identification of novel NAT10 inhibitors. Based on this information, we identified Fosaprepitant, Leucal, Fludarabine and Dantrolene as promising candidates that can be developed as potential inhibitors of NAT10 acetyltransferase activity.

## Materials and methods

### Computational simulations

The computer simulations were done in Maestro graphical user interphase of Schrödinger (www.schrodinger.com) on a laptop with (Ubuntu 20.04.1 LTS operating system, Graphics card NVIDIA GeForce RTX2070, CPU 2.20GHz*12, Intel Core i7, and RAM 15.5GiB, Disk Capacity 1.5 TB).

### Homology modeling

Homology modeling was performed to generate a three-dimensional (3D) structure of human N-acetyltransferase 10 (NAT10) protein. The Prime tool of the Maestro interface (Schrödinger Release 2020-3) was used for the model generation^45^. BLAST homology search in Prime interface was used for searching of a similar model in the PDB database. The tRNA (Met) Cytidine acetyltransferase (PDB ID: 2ZPA) model was used as a template for the structure prediction of NAT10. Then loops of the generated structure were refined using the Prime refine loops tool. Ramachandran plot in Maestro was used for estimation of built structure quality. Molecular Dynamics (MD) simulation run at 50 ns for calculation of Root-Mean-Square Deviation and Fluctuation (RMSD and RMSF).

### Protein preparation and active site prediction

The NAT10 structure was optimized using the Protein Prep Wizard tool in Maestro. Using the PROPKA tool, an ionization state was generated at pH 7.4, a network of hydrogen bonds was generated, and the overall NAT10 structure was minimized.

### Ligand preparation

Acetyl-CoA, Remodelin and FDA-approved drugs (2115) structures were downloaded from ZINC database. Acetyl-CoA, Remodelin and FDA-approved drugs were all optimized in LigPrep of Maestro software (Schrödinger Release 2020-3). Using LigPrep the 3D coordinates of all compounds were generated. The module Epik predicted the ionization state at pH 7.0–7.4. Other features such as chirality and tautomer forms were generated and properly defined. The geometries of all compounds were minimized using OPLS3e force-field.

### Molecular docking

To identify protein active site SiteMap and Receptor Grid Generation in Maestro were used for binding pocket search. We selected the binding pocket based on N-acetyltransferase amino acid residue sequence and position **(558–753)** as illustrated by Larrieu et al.^7^. Remodelin compound was used for comparison of the docking result^7^, using extra precision docking (XP-docking) mode. For FDA-approved drugs, High Throughput Virtual Screening (HTVS) mode was used to screen 2115 drugs, the top 200 shortlisted drugs based on docking score were then screened using Standard Precision mode (SP-docking). Finally, the top 50 molecules were screened using XP-docking. The molecular dynamic simulation was done on the complex of NAT10 and top 4 FDA-approved drugs in addition to Acetyl-CoA and Remodelin.

### Molecular dynamic simulations

Based on results obtained from molecular interaction and visual analysis for Acetyl-CoA, Remodelin and top 4 FDA-approved drugs screened by XP-docking and SP-docking. The complexes of these Ligands with NAT10 were considered for Molecular Dynamic Simulations (MDS) study. The MDS was run on Desmond Module (Schrödinger Release 2020–3) using the System Builder tool in Desmond; the complex was soaked into an orthorhombic box of 10 Å distance and filled with TIP4P waters. Then the system ions were neutralized. The complex was equilibrated at 300 K and 1 atmospheric pressure by NVT and NPT ensembles. Root-mean-square deviation and fluctuation (RMSD and RMSF) were then calculated.

### Multiple sequence alignment of N-acetyltransferase of NAT10

To carry out the multiple sequence alignment of N-acetyltransferase across different species. The N-acetyltransferase domain sequence was retrieved from Uniprot (https://www.uniprot.org/) for species such as *Homo sapiens* (Q9H0A0), *Rattus norvegicus* (G3V752), *Mus musculus* (Q8K224), *Drosophila melanogaster* (Q9W3C1), *Escherichia coli* (P76562), *Schizosaccharomyces pombe* (P87115), *Arabidopsis thaliana* (Q9M2Q4), *Dictyostelium discoideum* (Q55EJ3), *Caenorhabditis elegans* (O01757), *Haemophilus influenzae* (P44140), *Vibrio cholerae serotype O1* (Q9KKJ5), *Salmonella typhimurium* (Q8ZN74), *Yersinia pestis* (D0JFM7), and *Yersinia enterocolitica serotype O:8* (A1JL12). Retrieved sequences were aligned using BioEdit 7.0.9 software^46^.
